# MethPed: a DNA methylation classifier tool for the identification of pediatric brain tumor subtypes

**DOI:** 10.1186/s13148-015-0103-3

**Published:** 2015-07-09

**Authors:** Anna Danielsson, Szilárd Nemes, Magnus Tisell, Birgitta Lannering, Claes Nordborg, Magnus Sabel, Helena Carén

**Affiliations:** Department of Oncology, Sahlgrenska Cancer Center, Institute of Clinical Sciences, Sahlgrenska Academy, University of Gothenburg, Gothenburg, Sweden; Swedish Hip Arthroplasty Register, Centre of Registers Västra Götaland, Gothenburg, Sweden; Department of Clinical Neuroscience and Rehabilitation, Institute of Neuroscience and Physiology, Sahlgrenska Academy, University of Gothenburg, Gothenburg, Sweden; Department of Pediatrics, Institute of Clinical Sciences, Sahlgrenska Academy, University of Gothenburg, The Queen Silvia Children’s Hospital, Sahlgrenska University Hospital, Gothenburg, Sweden; Department of Pathology, Sahlgrenska University Hospital, Gothenburg, Sweden; Department of Pathology, Sahlgrenska Cancer Center, Institute of Biomedicine, Sahlgrenska Academy, University of Gothenburg, PO Box 425, SE-40530 Gothenburg, Sweden

**Keywords:** DNA methylation, 450 K, Random forest, PNET, GBM, Medulloblastoma, Ependymoma, Classifier (classification tool), Astrocytoma, MethPed

## Abstract

**Background:**

Classification of pediatric tumors into biologically defined subtypes is challenging, and multifaceted approaches are needed. For this aim, we developed a diagnostic classifier based on DNA methylation profiles.

**Results:**

Methylation data generated by the Illumina Infinium HumanMethylation 450 BeadChip arrays were downloaded from the Gene Expression Omnibus (*n* = 472). Using the data, we built MethPed, which is a multiclass random forest algorithm, based on DNA methylation profiles from nine subgroups of pediatric brain tumors. DNA from 18 regional samples was used to validate MethPed. MethPed was additionally applied to a set of 28 publically available tumors with the heterogeneous diagnosis PNET. MethPed could successfully separate individual histology tumor types at a very high accuracy (*κ* = 0.98). Analysis of a regional cohort demonstrated the clinical benefit of MethPed, as confirmation of diagnosis of tumors with clear histology but also identified possible differential diagnoses in tumors with complicated and mixed type morphology.

**Conclusions:**

We demonstrate the utility of methylation profiling of pediatric brain tumors and offer MethPed as an easy-to-use toolbox that allows researchers and clinical diagnosticians to test single samples as well as large cohorts for subclass prediction of pediatric brain tumors. This will immediately aid clinical practice and importantly increase our molecular knowledge of these tumors for further therapeutic development.

**Electronic supplementary material:**

The online version of this article (doi:10.1186/s13148-015-0103-3) contains supplementary material, which is available to authorized users.

## Background

Tumors of the central nervous system (CNS) are the most common solid malignancies in children, representing about 20 % of all childhood cancer cases [1]. Overall survival of children with brain tumors is around 70 % but varies highly depending on type and location of the tumor.

Classification of pediatric tumors into biological relevant entities is challenging and vitally important in determining the appropriate treatment protocol for a specific patient [2, 3]. Childhood cancer survivors often experience substantial long-term side effects from the treatment. Choosing the right treatment and avoiding unnecessary treatment is therefore very important. An appropriate reproducible classifier is thus urgently needed to define good and poor treatment response subgroups and for the evaluation of results obtained from clinical trials in order to validate the potency of new drugs specifically designed to selectively affect molecular targets in the respective subclasses.

The most common clinical diagnosis groups include pilocytic astrocytoma, high-grade glioma/glioblastoma (GBM), diffuse intrinsic pontine glioma (DIPG), ependymoma, and primitive neuroectodermal tumor of the CNS (CNS-PNET), medulloblastoma (cerebellar PNET), and supratentorial PNET (sPNET); however, there are more than 100 different histological subtypes. Using conventional parameters such as location and histology (WHO criteria) for diagnosis will not capture the full picture of these tumors and thus lead to both under- and overtreatment as well as hamper the identification of prognostic factors and molecular biomarkers [4].

Previous studies have shown that methylation profiling using the Illumina 450K methylation arrays can divide several pediatric brain tumor diagnoses including the four medulloblastoma subgroups; sonic hedgehog (MB_SHH), WNT (MB_WNT), group 3 (MB_Gr3), and group 4 (MB_Gr4) [5–9]. However, a classification tool for diagnosing an unknown tumor is still lacking. In the current study, we developed a classification tool, MethPed, which can robustly identify brain tumor diagnoses and subgroups using genome-wide DNA methylation array data, which outperforms previous methods using for example gene expression data [10].

## Results

In this study, publically available Illumina 450K methylation array data from 472 pediatric brain tumors, representing several diagnoses (DIPG, GBM, embryonal tumors with multilayered rosettes (ETMR), four medulloblastoma subgroups, ependymoma, and pilocytic astrocytoma) were used to build a diagnostic classifier.

### Building the DNA methylation classifier MethPed

**Fig. 1:**
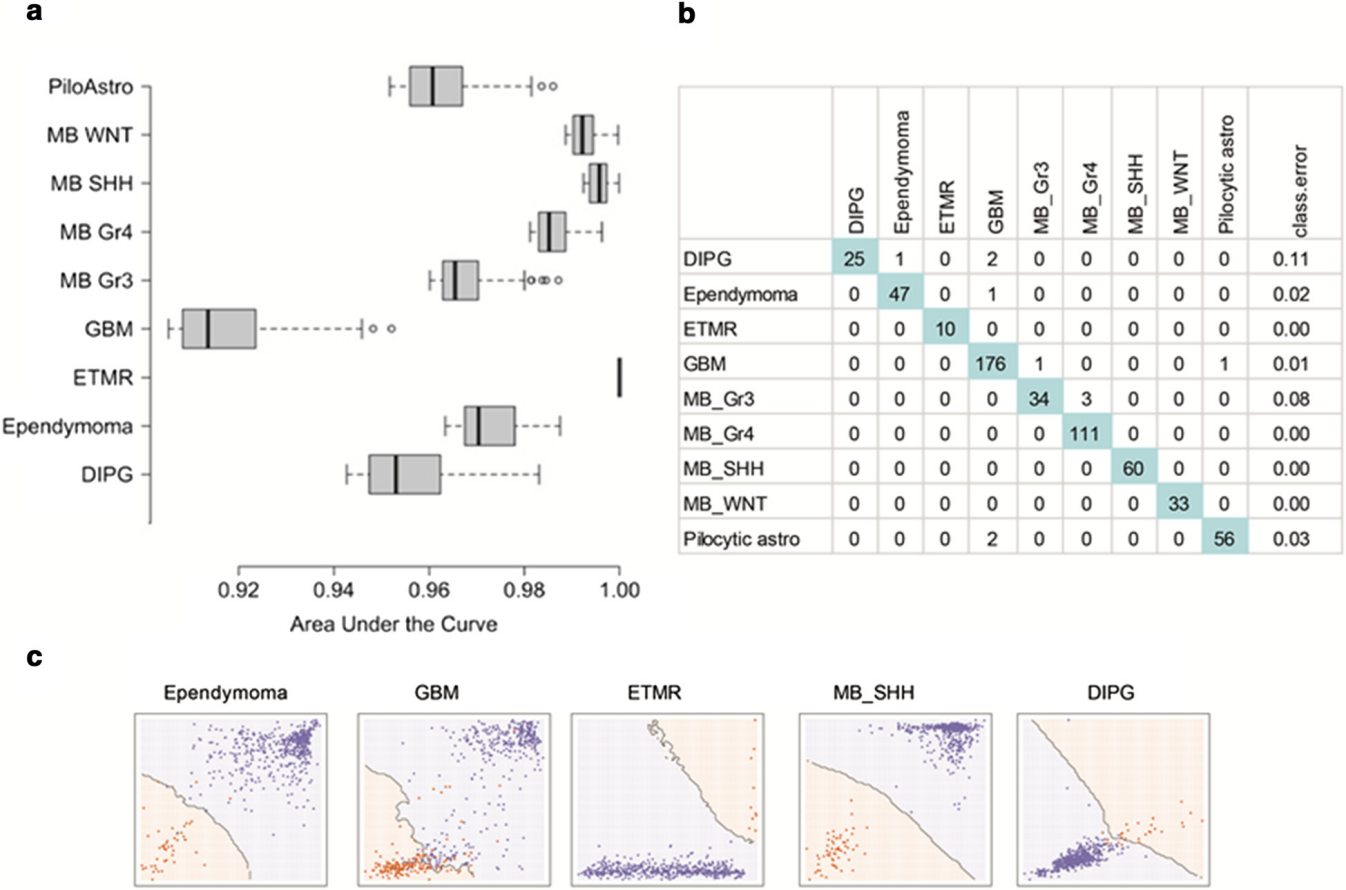
Accuracy of the MethPed classifier. **a** Classification accuracy of individual methylation probes in one vs all other logistic regression analyses. The *boxplots* displays the classification accuracy as measured by the area under the curve (AUC values or c-statistics) for the 100 probes per tumor subtype that provided the highest predictive power; **b** Confusion matrix showing an extremely high predictive capacity of MethPed, illustrated by the high percentage of correct classification of randomly drawn pairs; and **c** Decision boundaries for five tumor types exemplifying the possibility to delimitate a certain tumor type from the rest based on the two probes that proved to be the best for each tumor in one vs all other regression analyses

We used a large number of regression analyses to select the 100 probes per tumor class that had the highest predictive power. Thereafter, a Random Forest algorithm was fit to the data to develop the MethPed classifier. Individual methylation profiles could successfully separate distinct tumor types with high accuracy when one tumor was compared with all others. All sites had AUC values of more than 90 % and for most cases, offered almost prefect classification (Fig. 1a). Based on the 900 methylation sites (Additional file 1: Table S1), the nine pediatric brain tumor types could be accurately classified using the multiclass classification algorithm MethPed; the overall error rate was only 1.7 %. The tumor entities ETMR, MB_Gr4, MB_SHH, and MB_WNT were perfectly classified (Fig. 1b). Cohen’s Kappa statistic (0.978, 95 % CI, 0.972–0.983) were in agreement with the overall accuracy rate, indicating that the overall error rate is a fair estimate and is not a result of imbalances among the groups. For some tumor entities, even a couple of methylation sites offered very accurate classification. Figure 1c shows how the most differentially methylated CpG sites can delimitate a certain tumor type from the rest. For example, only two CpG sites offer full separation of the Shh group of medulloblastomas to the rest of the tumors, as is the case also for ETMR tumors. On the other hand, GBM tumors are more heterogeneous as a group and hence require more CpG sites for accurate separation.

### Analysis of a regional cohort

To test the MethPed in a clinical setting, we analyzed a consecutive set of 18 pediatric brain tumors obtained from the Sahlgrenska University Hospital, Sweden, between 2013 and 2014. The analysis of the regional cohort demonstrated the clinical benefit of MethPed, as it confirmed tumors with a straightforward diagnosis but also identified possible differential diagnoses in tumors with complicated and mixed type morphology. Three children in the cohort were diagnosed with glioblastoma according to the WHO criteria which was verified with MethPed (strength of 91, 85, and 64 % respectively; Table 1). Tumors with the diagnosis pilocytic astrocytoma were all classified with high probability as such. Two cases with the histopathological diagnosis sPNET (a diagnosis not included in MethPed) were assigned to the glioblastoma subclass, whereas the remaining one got an inclusive score. Among the four medulloblastomas, three could be further subgrouped into the relevant molecular medulloblastoma tumor groups, but one case did not share the methylation profile of any of the medulloblastoma groups (Table 1). This case was not classified robustly to any of the diagnostic groups in the classifier, suggesting that it is instead a rare tumor form.

**Table 1:** MethPed classification of the regional cohort

Patient ID	Histopathologic diagnosis (WHO criteria)	MethPed classification	DIPG	Ependymoma	ETMR	Glioblastoma	MB_Gr3	MB_Gr4	MB_SHH	MB_WNT	Pilocytic astrocytoma
BPC-B2	Glioblastoma	Glioblastoma	0.03	0.06	0.03	*0.64*	0.02	0.01	0.01	0.00	0.21
BPC-B0	Glioblastoma	Glioblastoma	0.01	0.03	0.00	*0.91*	0.00	0.00	0.01	0.00	0.05
BPC-C3	Glioblastoma	Glioblastoma	0.01	0.04	0.01	*0.86*	0.01	0.00	0.01	0.00	0.06
BPC-C8	Glioblastoma	Glioblastoma	0.01	0.05	0.01	*0.90*	0.00	0.00	0.01	0.00	0.03
BPC-A6	Glioneural tumor WHO grade IV	Glioblastoma	0.02	0.09	0.02	*0.61*	0.01	0.02	0.01	0.00	0.22
BPC-C5	Oligoastrocytoma WHO grade II	Glioblastoma	0.05	0.05	0.01	*0.75*	0.01	0.01	0.01	0.01	0.10
BPC-A4	Pilocytic Astrocytoma	Pilocytic astrocytoma	0.01	0.03	0.00	0.19	0.00	0.00	0.00	0.00	*0.77*
BPC-B6	Pilocytic Astrocytoma	Pilocytic astrocytoma	0.02	0.05	0.01	0.28	0.00	0.00	0.00	0.00	*0.63*
BPC-B8	Pilocytic Astrocytoma	Pilocytic astrocytoma	0.01	0.05	0.00	0.21	0.00	0.01	0.00	0.00	*0.72*
BPC-A8	Glioneuronal (pilocytic/pilomyxoid)	Pilocytic astrocytoma	0.02	0.03	0.02	0.24	0.01	0.00	0.00	0.00	*0.68*
BPC-B5	PNET	Glioblastoma	0.03	0.30	0.03	*0.58*	0.01	0.00	0.01	0.01	0.03
BPC-B7	PNET	Glioblastoma	0.05	0.09	0.03	*0.49*	0.01	0.01	0.00	0.01	0.32
BPC-A7	PNET	Glioblastoma	0.03	0.10	0.05	*0.74*	0.02	0.01	0.01	0.02	0.03
BPC-C1	Medulloblastoma (large-cell)	Mixed	0.06	0.21	0.19	0.32	0.03	0.04	0.04	0.05	0.06
BPC-C4	Medulloblastoma (desmoplastic)	MB_SHH	0.01	0.07	0.02	0.09	0.02	0.01	*0.74*	0.03	0.02
BPC-A	Medulloblastoma	MB_Gr4	0.01	0.02	0.00	0.02	0.14	*0.78*	0.01	0.02	0.00
BPC-B1	Medulloblastoma (classic)	MB_Gr4	0.00	0.03	0.01	0.01	0.06	*0.89*	0.01	0.01	0.01
BPC-A1	Medulloblastoma (classic)	MB_Gr3	0.01	0.04	0.01	0.02	*0.82*	0.08	0.01	0.01	0.01

**Fig. 2:**
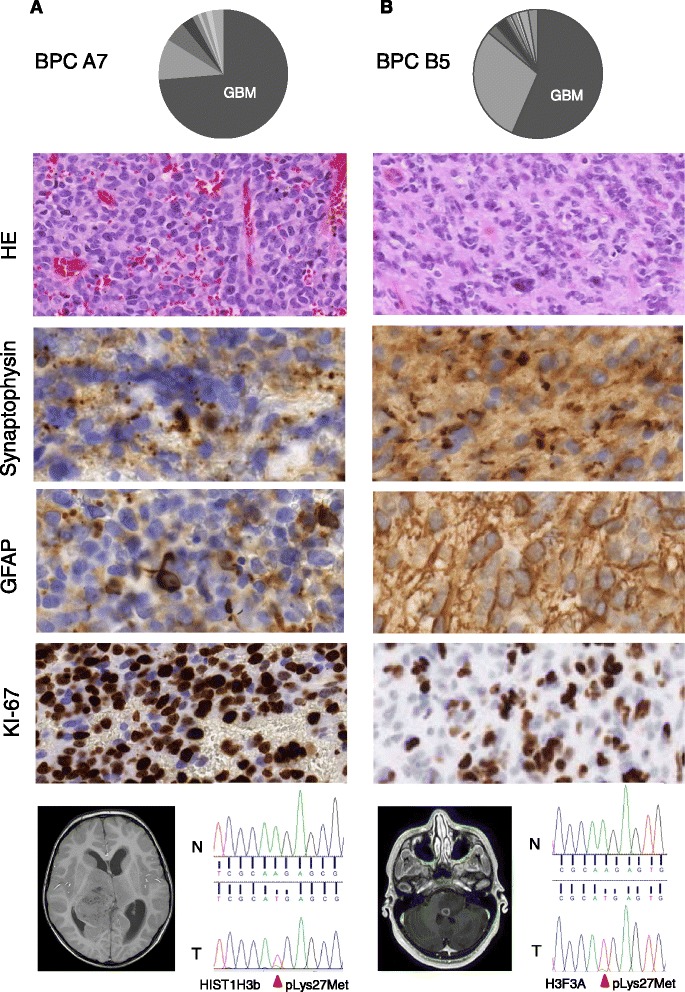
Histopathological and molecular analyses of two patients in the regional cohort. **a** Four-year-old child (BPC A7) diagnosed with a PNET in the right hemisphere. MethPed classification (*upper panel*). H&E shows polymorphic, anaplastic cells and regions with necrotic areas; synaptophysin shows clonal positivity; GFAP mostly negative areas but also individual tumor cells with very strong expression and Ki-67 variable positivity (*middle section*, original magnification of the objective in all cases ×40). Magnetic resonance imaging (MRI) shows the location of the tumor, and Sanger sequencing chromatogram shows a HIST1H3B Lys27Met mutation in the tumor. *Red arrow* indicates the site of the mutation (*lower panel*). **b** Twelve-year-old child (BPC B5) diagnosed with a PNET in the brain stem. MethPed classification (*upper panel*). H&E shows cells variable in morphology with areas of rosette formation similar to Homer-Wright type; synaptophysin areas with granular cytoplasmic pattern and other areas with diffuse positivity as well as negative cells; GFAP positivity in a high number of cells indicates an unusual high incidence of astrocytic differentiation and high positivity of Ki-67 (*middle section*, objective original magnification ×40). Magnetic resonance imaging (MRI) shows the location of the tumor, and Sanger sequencing chromatogram shows a H3F3A Lys27Met mutation in the tumor. *red Arrow* indicates the site of the mutation (*lower panel*)

**Fig. 3:**
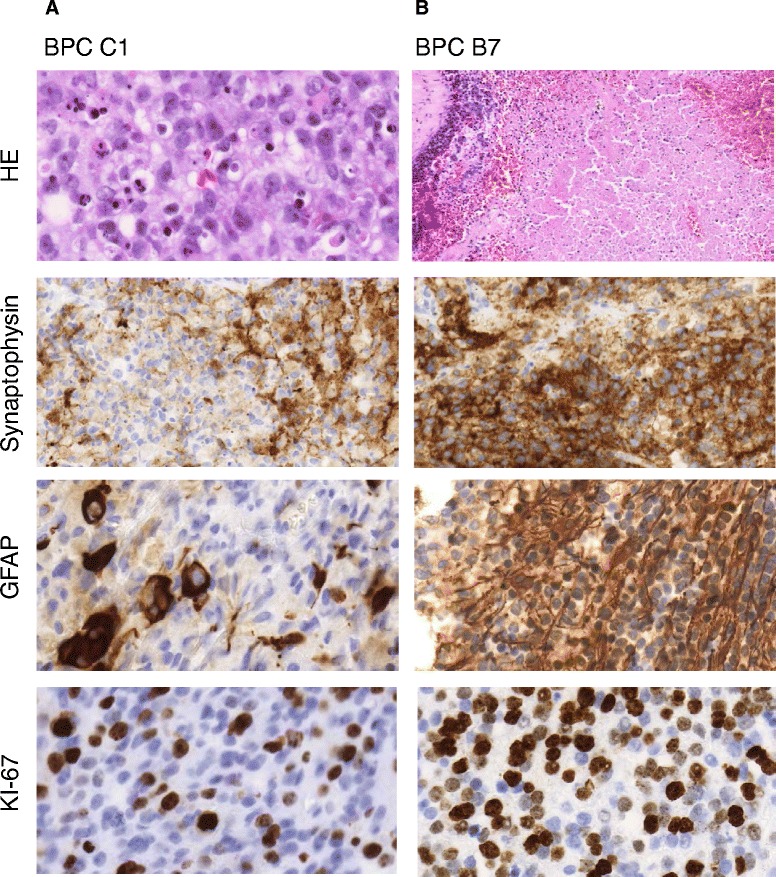
Immunohistochemical analyses of two patients with challenging diagnoses. **a** Infant (case BPC C1) diagnosed according to the WHO criteria with a large-cell medulloblastoma, located in the vermis. H&E shows predominantly large cells with a high frequency of apoptotic bodies, clonal positivity of GFAP, and low positivity for synaptophysin and clonal areas with high Ki-67 positivity (objective original magnification × 40). **b** Four-year-old child (BPC B7) diagnosed with an intra- and periventricular PNET tumor. H&E shows high frequency of necrosis and vessels, very strong, clonal positivity of GFAP in tumor cells as well as positivity in reactive gliosis, high positivity for synaptophysin, and high Ki-67 positivity (objective original magnification ×40)

To scrutinize the discrepancy between MethPed and the histopathological diagnosis, these cases were reviewed by a senior neuropathologist who re-evaluated the original paraffin HE histology, the immunohistochemical staining of neurons with the presynaptic marker synaptophysin (SYP), astrocytic marker glial fibrillary acidic protein (GFAP), and the marker of proliferation, Ki-67 (MKI67) (Fig. 2a, b and Fig. 3a, b). Furthermore, we performed mutation analysis which confirmed histone mutations at Lys27Met at H3F3A and H1H3b in both cases with the histopathological diagnosis sPNET, assigned as GBMs by MethPed (Table 1 and Fig. 2a, b). In addition, these tumors showed aggressive clinical behavior with resistance to therapy.

### Applying the MethPed algorithm to a heterogeneous WHO diagnosis

The finding that the PNET samples in our regional cohort was classified as GBMs prompted us to analyze this group of tumors more closely. For this aim, we used a publically available data set composed of 28 PNET tumors (GEO accession GSE52556) [11]. MethPed could, with a high accuracy, classify many of these tumors as GBMs, ependymomas, or one of the medulloblastoma subgroups, demonstrating the benefit of using the MethPed classifier for identifying more likely diagnoses (Table 2).

**Table 2:** MethPed classification of a set of PNET cases

Case ID	DIPG	Ependymoma	ETMR	Glioblastoma	MB_Gr3	MB_Gr4	MB_SHH	MB_WNT	Pilocytic astrocytoma
PNET 1	0.02	0.04	0.05	*0.85*	0.01	0.01	0.01	0.00	0.03
PNET 2	0.05	0.09	0.14	*0.46*	0.08	0.08	0.01	0.04	0.04
PNET 3	0.02	0.02	0.01	*0.91*	0.00	0.01	0.00	0.00	0.03
PNET 4	0.03	0.07	0.02	*0.63*	0.02	0.01	0.01	0.02	0.21
PNET 5	0.01	0.03	0.01	*0.79*	0.03	0.02	0.03	0.01	0.08
PNET 6	0.01	0.01	0.01	*0.91*	0.01	0.00	0.00	0.00	0.05
PNET 7	0.05	0.19	0.04	*0.54*	0.03	0.02	0.04	0.02	0.08
PNET 8	0.07	*0.50*	0.07	0.27	0.03	0.02	0.01	0.02	0.02
PNET 9	0.04	0.08	0.14	*0.40*	0.08	0.05	0.01	0.06	0.15
PNET 10	0.04	0.09	0.08	*0.49*	0.08	0.06	0.03	0.03	0.11
PNET 11	0.04	0.12	0.04	*0.47*	0.05	0.04	0.02	0.02	0.21
PNET 12	0.06	*0.48*	0.07	0.27	0.05	0.03	0.01	0.02	0.02
PNET 13	0.05	0.05	0.02	*0.68*	0.01	0.01	0.00	0.01	0.18
PNET 14	0.06	*0.44*	0.05	0.36	0.02	0.02	0.02	0.01	0.03
PNET 15	0.02	0.11	0.05	*0.57*	0.07	0.03	0.04	0.04	0.08
PNET 16	0.01	0.02	0.03	0.07	0.21	0.04	0.00	*0.60*	0.03
PNET 17	0.00	0.00	0.00	0.01	0.04	*0.94*	0.00	0.00	0.00
PNET 18	0.01	0.02	0.02	0.01	0.03	0.02	*0.88*	0.00	0.01
PNET 19	0.07	0.03	0.03	*0.81*	0.01	0.02	0.01	0.01	0.02
PNET 20	0.06	0.04	0.07	0.10	0.08	0.07	*0.53*	0.02	0.03
PNET 21	0.03	0.04	0.04	0.07	*0.63*	0.08	0.01	0.07	0.03
PNET 22	0.00	0.00	0.00	0.00	0.08	*0.90*	0.00	0.01	0.00
PNET 23	0.09	0.09	0.16	*0.46*	0.07	0.05	0.01	0.02	0.05
PNET 24	0.05	0.31	0.03	*0.50*	0.01	0.01	0.01	0.01	0.08
PNET 25	0.11	0.13	0.07	*0.58*	0.02	0.02	0.03	0.01	0.03
PNET 26	0.02	0.06	0.02	*0.70*	0.04	0.02	0.02	0.02	0.11
PNET 27	0.01	0.02	0.01	0.03	0.01	0.01	*0.86*	0.02	0.01
PNET 28	0.00	0.01	0.00	0.02	0.01	0.00	*0.96*	0.00	0.00

## Discussion

Stratification of patients with pediatric tumors with differing biological behavior or responsiveness to specific therapies is urgently needed. Molecular subgrouping has been documented as a useful clinical tool. We therefore built a robust classifier using DNA methylation profiles that could successfully classify pediatric brain tumors into clinically relevant subgroups. We included the most common brain tumors in children in MethPed, as well as the very rare tumor ETMR as the incidence of this often misdiagnosed tumor is thought to be underestimated. MethPed performed well both in internal and external validation and is novel as it can classify different diagnoses and is therefore not limited to subgroup classification. The MethPed classification tool outperforms previously published classifiers using differentially expressed genes as input and those that only handle medulloblastoma subgroups [10, 12].

The accuracy of the MethPed classifier was further corroborated by classifying a new cohort of 18 pediatric brain tumors and by matching the classification results with the histopathological diagnoses according to WHO. With the increased knowledge about specific brain tumor subgroups and the development of targeted therapy for different entities, it is now very important to accurately determine the correct diagnosis for this group of patients. Importantly, as pediatric brain tumors are rare and the experience in diagnosing them varies among hospitals and countries, MethPed provides an independent tool.

Here, we included nine tumor types in MethPed, but the method can be further developed to incorporate additional tumor types. The applied Random Forests method can be extended when additional data sets become available as it is efficient with large data sets and does not overfit the data. Methylation profiles are considered stable, and through logistic regression, a set of probes within each class were identified which gave high accuracy in prediction. Compared to hierarchical clustering methods, MethPed enable classification of single samples as generated forests can be saved for future use on other data.

CNS-PNET is an embryonal neoplasm with medulloblastoma-like histology; the current WHO criterion does not distinguish CNS-PNETs in the form of medulloblastoma in the cerebellum or in the form of a supratentorial PNET. However, recent studies have shown that histologically defined CNS-PNETs display heterogeneous methylation profiles and show relationships to other pediatric brain tumor types [12]. Thus, a high frequency of PNETs might be misdiagnoses of other tumor forms, and new criteria for diagnosing true CNS-PNET tumors are therefore needed, which is why we did not include the current PNET diagnosis group in MethPed. To illustrate the heterogeneous profiles of PNETs, we ran a set of 28 CNS-PNET tumors through MethPed. Many of the samples could be accurately classified into one of the nine diagnoses/subgroups in MethPed, whereas some could not confidently be classified into either of these, suggesting that they are true PNETs or alternatively other rare tumors. Pediatric GBMs have been reported to have a distinctive molecular pathogenesis with high frequency of H3F3A mutations; thus the histone mutations present in the two regional PNET cases classified as GBM by MethPed support our results [4, 13]. We next re-examined the histopathological material from these cases and found focal areas of differentiated cells indicative of GBM. High-grade gliomas such as GBM typically arise from astrocytic origins, while CNS-PNET is of predominantly neuronal origin, with medulloblastoma-like histology. Based on genetic and histology data, Perry et al. suggested that PNET- like nodules may arise in a preexisting glioma, most often a GBM [14]. Our reclassification results identified diagnostic pitfalls and highlights that cells with DNA methylation pattern of glioblastoma features may be seen in tumors of different histological types from different anatomical sites. Importantly, the diagnosis GBM instead of a PNET would change the treatment protocol for the patient. Additionally, it is important to identify tumors with mixed cell populations when planning an optimal treatment regime for a specific patient [15].

## Conclusions

We have developed the MethPed classifier that predicts brain tumor subtypes with a very high accuracy. The present tool will clinically aid to efficiently categorize the tumors of newly diagnosed patients, aid in choosing patients for clinical trials of newly developed targeted therapy, and aid to give insights into the underlying biology of the specific groups.

## Methods

### Data sets

Methylation data generated by the Illumina Infinium HumanMethylation 450 BeadChip arrays were downloaded from the Gene Expression Omnibus (GEO). Four hundred seventy-two cases were available, representing several brain tumor diagnoses (DIPG, GBM, ETMR, medulloblastoma, ependymoma, pilocytic astrocytoma) and their further subgroups (Table 3). The data sets were merged, and probes that did not appear in all data sets were filtered away. In addition, about 190,000 CpGs were removed due to SNPs, repeats, and multiple mapping sites [16]. The final data set contained 206,823 unique probes. K–neighbor imputation was used to deal with missing probe data [17].

**Table 3:** Data sets used in the study

Diagnosis	GEO accession	Citation
DIPG	GSE50022	[21]
Glioblastoma	GSE55712, GSE36278	[22, 23]
ETMR	GSE52556	[11]
Medulloblastoma	GSE54880	[24]
Ependymoma	GSE45353	
Pilocytic astrocytoma	GSE44684	[25]

### Verification set

DNA from 16 fresh frozen tissues and 2 paraffin embedded (FFPE) sample was used to validate MethPed. The tumor samples were obtained after signed informed consent from the parents of children who underwent surgery at the Sahlgrenska University Hospital, and the study was approved by the regional ethics committee (Dnr 604–12). Using the EZ DNA methylation kit (D5001, Zymo Research), 500 ng of DNA was bisulfite converted and hybridized to the Infinium HumanMethylation450 BeadChips (Illumina). The data generated by the BeadStudio software was exported and further analyses were performed in the R software environment. For this set of tumors, complete clinical information, including the histologic assessment, tumor sections, and frozen material, were available. In addition, 28 publically available tumors (GEO accession GSE52556) were used to specifically apply MethPed on tumors diagnosed as PNET [11].

### Computational analysis

The computational process proceeded in two stages. The first stage commenced with a reduction of the probe pool. A series of one vs all other logistic regression classifiers were run for each tumor type. The measure of interest was the classifiers predictive capacity as summarized by the area under the curve (Fig. 1a). For each tumor type, we ran 206,823 regression analyses. This stage ended with the selection of 100 probes per tumor class that had the highest predictive power. Thereafter, a Random Forest (RF) algorithm was fit to the data [18, 19]. Random Forest pools together many noisy but approximatively unbiased models, hence, reducing the predictions variance. The working model of the Random Forest algorithm is a simple classification tree. Random forest aggregates a predefined number of trees (900 in our case). At first, a bootstrap sample is drawn from the original data set, and a tree is trained on this bootstrap sample using only a subset of randomly selected predictors. The ideal number of predictors used for tree training cannot be estimated from the data and acts as a tuning parameter. We used grid search to find the ideal number of probes. Every tree assigns a class belonging to each tumor considered. The final classification is simply the majority vote. The probability of belonging to one or the other class is the number of votes each class receives divided by the number of trees grown. Validation proceeded with 10-fold cross-validation, repeated five times. We used the Kappa statistics as accuracy measurement which relates the observed accuracy to the accuracy that would be generated by simple chance [20]. Accuracy measurement was estimated on the out-of-bag samples only. In addition to Random Forest, other classification algorithms were tested as well, among other variations of discriminant analysis and Stochastic Generalized Boosted Models. However, these models either had lower or similar performance but at the price of substantially higher computational burden. The MethPed classifier uses the Random Forest algorithm to classify new tumors pediatric brain tumor subtypes. The classification proceeds with the selection of the methylation probes needed for the classification. Thereafter, based on the built algorithm, a conditional probability of pediatric brain tumor subtypes belonging is calculated. For the practicalities of implementation, we refer the reader to the online supplemental material.

## Additional file

Additional file 1: Table S1. CpG sites in the MethPed classifier.
